# Secretory phospholipase A_2_-IIA overexpressing mice exhibit cyclic alopecia mediated through aberrant hair shaft differentiation and impaired wound healing response

**DOI:** 10.1038/s41598-017-11830-9

**Published:** 2017-09-14

**Authors:** Gopal L. Chovatiya, Rahul M. Sarate, Raghava R. Sunkara, Nilesh P. Gawas, Vineet Kala, Sanjeev K. Waghmare

**Affiliations:** 1 Stem Cell Biology Group, Waghmare Lab, Cancer Research Institute, Advanced Centre for Treatment Research and Education in Cancer (ACTREC), Tata Memorial Centre, Kharghar, Navi Mumbai, 410210 MH India; 2 Homi Bhabha National Institute, Training School Complex, Anushakti Nagar, Mumbai, 400085 India

**Keywords:** Differentiation, Differentiation, Skin stem cells, Skin stem cells, Skin stem cells

## Abstract

Secretory phospholipase A_2_ Group-IIA (sPLA_2_-IIA) is involved in lipid catabolism and growth promoting activity. sPLA_2_-IIA is deregulated in many pathological conditions including various cancers. Here, we have studied the role of sPLA_2_-IIA in the development of cyclic alopecia and wound healing response in relation to complete loss of hair follicle stem cells (HFSCs). Our data showed that overexpression of sPLA_2_-IIA in homozygous mice results in hyperproliferation and terminal epidermal differentiation followed by hair follicle cycle being halted at anagen like stage. In addition, sPLA2-IIA induced hyperproliferation leads to complete exhaustion of hair follicle stem cell pool at PD28 (Postnatal day). Importantly, sPLA_2_-IIA overexpression affects the hair shaft differentiation leading to development of cyclic alopecia. Molecular investigation study showed aberrant expression of Sox21, Msx2 and signalling modulators necessary for proper differentiation of inner root sheath (IRS) and hair shaft formation. Further, full-thickness skin wounding on dorsal skin of K14-sPLA_2_-IIA homozygous mice displayed impaired initial healing response. Our results showed the involvement of sPLA_2_-IIA in regulation of matrix cells differentiation, hair shaft formation and complete loss of HFSCs mediated impaired wound healing response. These novel functions of sPLA_2_-IIA may have clinical implications in alopecia, cancer development and ageing.

## Introduction

Skin constantly renews itself throughout the adult life that acts as a protective barrier against pathogens, radiation etc.^1^. Adult skin is mainly composed of epidermis, dermis and hypodermis. Epidermal components mainly include interfollicular epidermis (IFE), hair follicle and sebaceous gland^2^. Dermis and hypodermis are mainly composed of collagen, elastic fibers and extrafibrillar matrix with various cell types including fibroblasts, adipose cells and macrophages. During embryogenesis, hair follicle is formed as an appendage of the epidermis by condensation of specialized mesenchymal cells (dermal papilla) in the dermis. The basal layer cells overlying mesenchymal cells get stimulated and subsequently form placode at E14.5 that proliferate and grow downward as a mature hair follicle at E16.5-E17.5^3^. The hair follicle cycle comprises of distinct stages such as telogen (resting phase), anagen (growth phase) and catagen (regression phase)^4^. Initially, both the pulse-chase studies carried out using tritiated thymidine (^3^H) and BrdU showed the presence of infrequently dividing cells in the bulge of hair follicle^5^. Subsequently, pTre-H2BGFP/K5tTa (Tet off) double transgenic mice study showed that the hair follicle stem cells are highly dynamic, which divide infrequently and undergo random chromosome segregation to maintain tissue homeostasis^6–8^. During follicle regeneration, the dermal papilla at the base of hair follicle provides initiatory signalling cues generated from the mesenchymal niche^9^. The cyclic growth of hair follicle is coordinated by the stem cells residing at the base of the bulge, which proliferate and migrate to provide progeny required for the hair-follicle regeneration and hair growth^10^. Further, the temporal activity of hair follicle stem cells is strictly dependent on interplay between mesenchymal niche and bulge, which is governed by secretion of various signalling modulators such as Wnt, BMP, Shh, and FGF^11^. In particular, Wnt signalling is necessary for the hair follicle morphogenesis and required for stem cell proliferation and differentiation during hair follicle regeneration^12–14^. Additionally, EGF induced EGFR signalling is indispensable for the initiation of hair growth^15^. The EGF signalling modulator, secretory phospholipase A_2_ group IIA (sPLA_2_-IIA) is also known as enhancing factor (EF), which expressed by paneth cells in the small intestine^16, 17^. sPLA_2_-IIA has two independent activities, catalytic and non-catalytic (enhancing) and both these activities rely on the two different domains of this enzyme^18^. Also, various studies have reported the expression of sPLA_2_-IIA in mouse epidermis^19, 20^. Moreover, sPLA_2_-IIA has been implicated in various forms of cancer such as intestinal, colorectal, prostrate^21^ and overexpression of sPLA_2_-IIA in mice epidermis showed increased susceptibility towards chemical carcinogenesis^22^. We have recently reported that sPLA_2_-IIA enhances the expression of Hb-EGF, EPGN and downstream c-Jun and Fos-B^23^. However, the molecular insight, if sPLA2-IIA regulates the hair shaft differentiation and the development of alopecia is still unknown. Secondly, do cells of other epidermal components form hair in the complete absence of hair follicle stem cells? Thirdly, whether wound healing process is impaired in the absence of HFSCs is yet to be determined.

Notably, deregulation of various signalling modulators perturbs stem cells maintenance that may results in development and progression of cyclic alopecia. Impaired EGFR signalling by dominant negative mutant of epidermal growth factor receptor in the epidermis prevents the progression of the hair cycle to catagen stage and causes severe alopecia^24^. Further, knockout of Sox21 in mice alters differentiation of cuticle layer leading to development of cyclic alopecia^25^. Also, epidermal ablation of Smad4 resulted in hyperplasia of interfollicular epidermis (IFE) and sebaceous glands (SGs), that leads to exhaustion of the SC niche and progressive hair loss^26^. Expression of noggin in epidermis resulted in upregulated Wnt signalling with epidermal hyperplasia, progressive hair loss, and formation of trichofolliculoma-like tumors^27^. Msx2 deficiency exhibits abnormal structure of hair shafts and cycles of hair loss and regrowth^28^. Targeted disruption of Orai1 gene in mouse showed sporadic hair loss while inducible deletion of cnB1 gene in mice resulted in altered hair follicle structure and its mesenchyme adhesion, that causes cyclic alopecia^29, 30^. However, the molecular mechanism involved in cyclic alopecia is yet to be discovered.

In this report, we studied the effect of K14-sPLA_2_-IIA expression in homozygous mice on alopecia and wound healing. To our knowledge, for the first time our findings on K14-sPLA_2_-IIA homozygous mice showed hair loss that is associated with an increased proliferation and differentiation of hair follicle stem cells, which led to exhaustion of hair follicle stem cells and development of cyclic alopecia at an early age. Additionally, K14-sPLA_2_-IIA homozygous mice showed impaired healing response during full thickness epidermal wounding.

## Results

### Altered hair follicle development and hair cycling in K14-sPLA_2_-IIA homozygous mice

**Figure 1:**
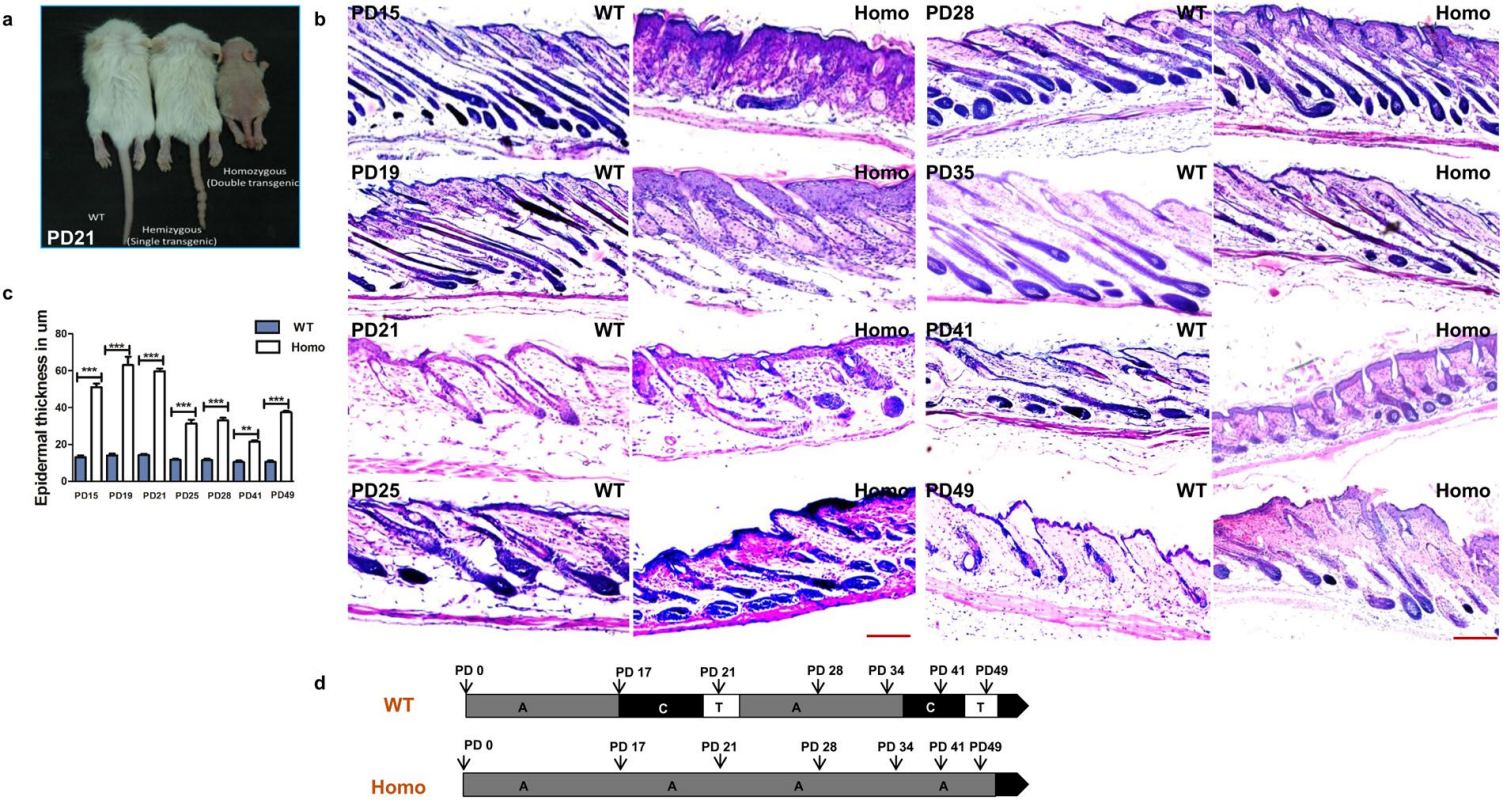
sPLA_2_-IIA overexpression altered the hair cycle with abnormal hair follicle morphology and hair loss. (**a**) Phenotypic appearance of WT, hemizygous and homozygous mice at PD21. (**b**) Images represents hematoxylin and eosin staining (H&E) of skin sections at various postnatal days of WT and K14-sPLA_2_-IIA homozygous mice to study hair follicle cycling. (**c**) Graphical representation of epidermal thickness measurements are in μm. Data are presented as mean ± SD. **P < 0.005, ***P < 0.001, ****P < 0.0001. (**d**) Comparative analysis of hair follicle progression to different stages of hair cycle (Anagen, Catagen and Telogen) at different postnatal days in WT and K14-sPLA_2_-IIA homozygous mice. (WT-Wild type, Homo- K14-sPLA_2_-IIA homozygous mice, n = 3 mice/genotype. A-Anagen, C-Catagen and T-Telogen, PD-Postnatal days).

To check the expression pattern of sPLA_2_-IIA in mice epidermis, we performed the IHC staining of sPLA_2_-IIA on skin sections of wild type FVB mice at various postnatal ages. Our data showed that sPLA_2_-IIA expresses in basal layer, suprabasal layer and outer root sheath of hair follicle during morphogenesis, first hair cycle and one year old age (Supplementary Fig. S1). K14-sPLA_2_-IIA homozygous mice exhibited visible phenotypic growth abnormalities and are significantly smaller than the control littermates (Fig. 1a). Further, to confirm the nutritional status of the K14-sPLA_2_-IIA homozygous mice, we have quantified various nutritional parameters from the serum of the K14-sPLA_2_-IIA homozygous mice. Our data showed that there are no significant alterations in serum components such as serum albumin, Vitamin D 25-OH, Triglyceride, Sodium, Chloride, and marginal increase was observed in total protein, Vitamin B12, Calcium and Potassium (Supplementary Fig. S4). However, we have observed reduced serum glucose level after eight hours fasting in the K14-sPLA_2_-IIA homozygous mice (Supplementary Fig. S4). These results demonstrate that there are no significant alterations in the nutritional parameters of the K14-sPLA_2_-IIA homozygous mice. These K14-sPLA_2_-IIA homozygous mice showed progressive hair loss during hair follicle morphogenesis periods (PD15 and PD19). Further, haematoxylin and eosin staining (H&E) on the dorsal skin sections was performed during various postnatal days (PD15, 19, 21, 25, 28, 35, 41 and 49) (Fig. 1b). Our histological data revealed interfollicular epidermal cyst formation and abnormal thickening of the interfollicular epidermis (IFE) (Fig. 1c) as compared to wild type control littermate. Further, hair follicle cycling analysis on the dorsal skin at various postnatal days showed hair follicle is halted at anagen like stage in K14-sPLA_2_-IIA homozygous mice (Fig. 1d). This is due to the fact that we have not observed telogen at any postnatal day (PD15, PD17, PD21, PD25, PD28, PD30, PD35, PD41, PD45 and PD49). The change in morphology of the hair follicle is due to abnormal development of epidermal compartments. Further, we observed increased activation of β-catenin in K14-sPLA_2_-IIA homozygous mice skin as compared to hemizygous and wild type control littermate (Supplementary Fig. S2). These data indicate that the hair follicles of K14-sPLA_2_-IIA homozygous mice failed to progress into regression phase (Catagen) of the hair follicle cycle. Thus, epidermal overexpression of sPLA_2_-IIA results in morphological abnormalities of hair follicle with hair loss.

### Abnormal organization of epidermal components and stratification of epidermis

**Figure 2:**
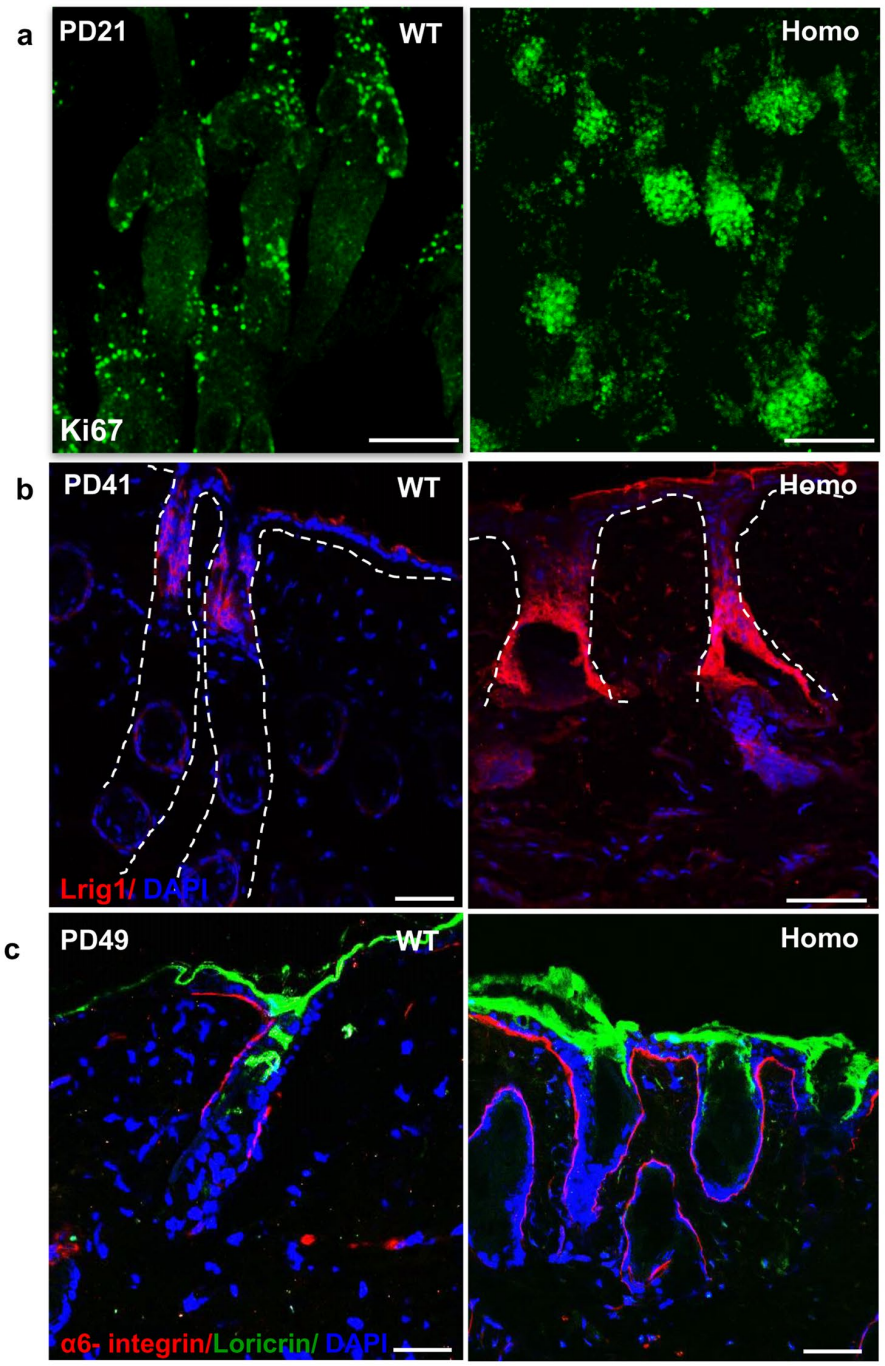
sPLA_2_-IIA induced proliferation affects various epidermal compartments. (**a**) Immunofluorescence staining of Ki67 to assess cell proliferation in intact epidermal sheet of tail skin by wholemount assay at PD21. (**b**) Immunofluorescence labelling of Lrig1 in WT and K14-sPLA_2_-IIA homozygous mice skin sections at PD41. Dashed lines represent the boundary of junctional zone area. (**c**) Immunofluorescence staining of loricrin as a differentiation marker to label the cells of granular layers in WT and K14-sPLA_2_-IIA homozygous mice at PD49.

Morphological abnormalities of the hair follicle led us to investigate if there is any effect on proliferation and differentiation in different epidermal compartments. To evaluate the effect of sPLA_2_-IIA on cell proliferation, we have performed immunofluorescence staining of Ki67, a proliferation marker on wholemount of intact epidermal sheets (Fig. 2a). Our results showed increased number of Ki67 positive cells in the outer root sheath and dermal papillae of the hair follicles, suggesting enhanced proliferation in K14-sPLA_2_-IIA homozygous mice skin. Moreover, we observed enlarged width of infundibulum and junctional zone regions of the hair follicle in K14-sPLA_2_-IIA homozygous mice. Therefore, we sought to check the expression of Lrig1, the marker of junctional zone stem cells by immunofluorescence staining on skin section, which showed significant increase in the Lrig1 positive cells (Fig. 2b). Further, to study whether the hyper-proliferative cells of IFE also generates more differentiated progeny, the later stages of terminal differentiation was evaluated. Immunofluorescence staining of Loricrin showed enhanced expression of Loricrin in K14-sPLA_2_-IIA homozygous mice skin, which indicates that sPLA2-IIA markedly increased epidermal differentiation (Fig. 2c). This result was further confirmed by the Real time quantitative analysis of S100a9 mRNA expression that showed drastic upregulation, suggesting increased differentiation in K14-sPLA_2_-IIA homozygous mice (Supplementary Fig. S3).

### Complete exhaustion of hair follicle stem cells in K14-sPLA_2_-IIA homozygous mice

**Figure 3:**
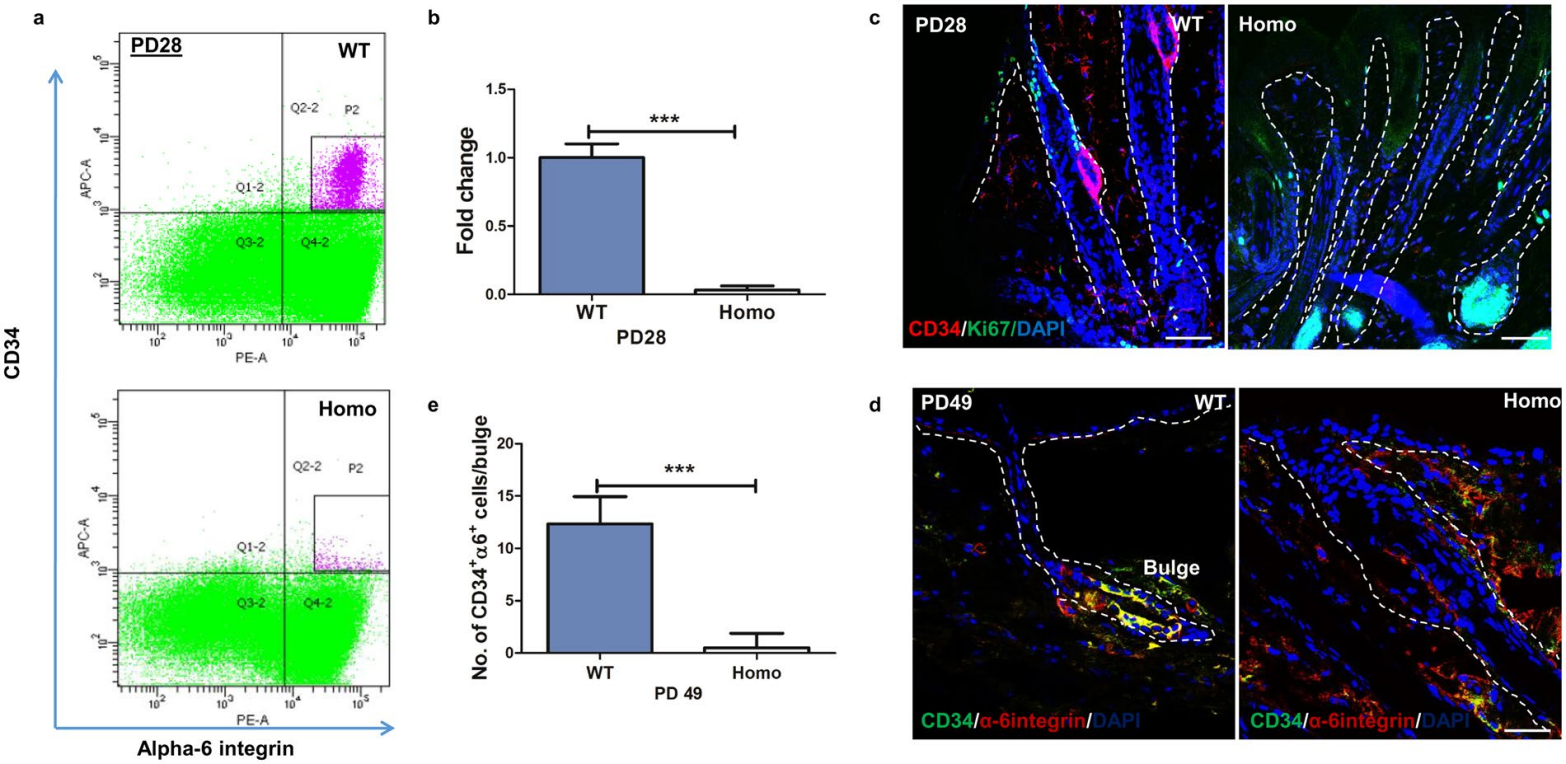
Overexpression of sPLA_2_-IIA leads to depletion of hair follicle stem cells. (**a**) Flow cytometry based analysis of hair follicle stem cells (CD34+/α-6 integrin+) in WT and K14-sPLA_2_-IIA homozygous mice at PD28. (**b**) Quantification of FACS analysis of CD34^+^/α6-integrin^+^ bulge HFSCs in wild type and K14-sPLA_2_-IIA homozygous mice at PD28. (**c**) Immunofluorescence analysis of CD34 and Ki67 expression in hair follicle at PD28. Scale bar: 50 µm. (**d**) Immunofluorescence analysis of CD34^+^/α6-integrin^+^ dual positive cells in hair follicle at PD49. Scale bar: 50 µm. (**e**) Quantification of CD34^+^/α6-integrin^+^ cells in the bulge of the dorsal skin in wild type and K14-sPLA_2_-IIA homozygous mice at PD49. PD-Postnatal days, HFSCs-Hair follicle stem cells. (WT-Wild type Homo- K14-sPLA_2_-IIA homozygous mice, n = 3 mice/genotype, PD-Postnatal days. Data are presented as mean ± SD. **P < 0.005, ***P < 0.001, ****P < 0.0001).

In K14-sPLA_2_-IIA homozygous mice, hair follicle was halted at anagen like stage and hair loss was observed with increase in proliferation and differentiation. Therefore, we attempted to understand the profile of hair follicle stem cells. We performed FACS analysis by using the mouse hair follicle stem cell markers such as CD34 and α6 integrin. Our results showed drastic depletion of CD34^+^/α6 integrin^+^ hair follicle stem cells at PD28 (Fig. 3a,b). Further, this data was validated by immunofluorescence staining (IFA) of CD34 and α6 integrin on the dorsal skin tissue sections at PD28 and PD49, which showed decrease in the number of CD34^+^/α6 integrin^+^ cells in hair follicle bulge of K14-sPLA_2_-IIA homozygous mice (Fig. 3c,d). Moreover, the counting of CD34/α6-integrin dual positive cells per hair follicle bulge showed complete loss of hair follicle stem cells at PD49 (Fig. 3e). In addition, to further confirm the loss of the hair follicle stem cells compartment, we have checked the Sox9 positive hair follicle stem cells in bulge of the hair follicle. Our data showed an absence of Sox9 positive cells in bulge region of hair follicle in the K14-sPLA2-IIA homozygous mice as compared to the wild type control littermate (Supplementary Fig. S5). This clearly demonstrates that the hair follicle stem cells pool is depleted. These data suggest that sPLA_2_-IIA induced hyper-proliferative response may lead to the subsequent complete exhaustion of hair follicle stem cell pool and aging-like skin phenotype in K14-sPLA_2_-IIA homozygous mice.

### Development of cyclic alopecia in K14-sPLA_2_-IIA homozygous mice

**Figure 4:**
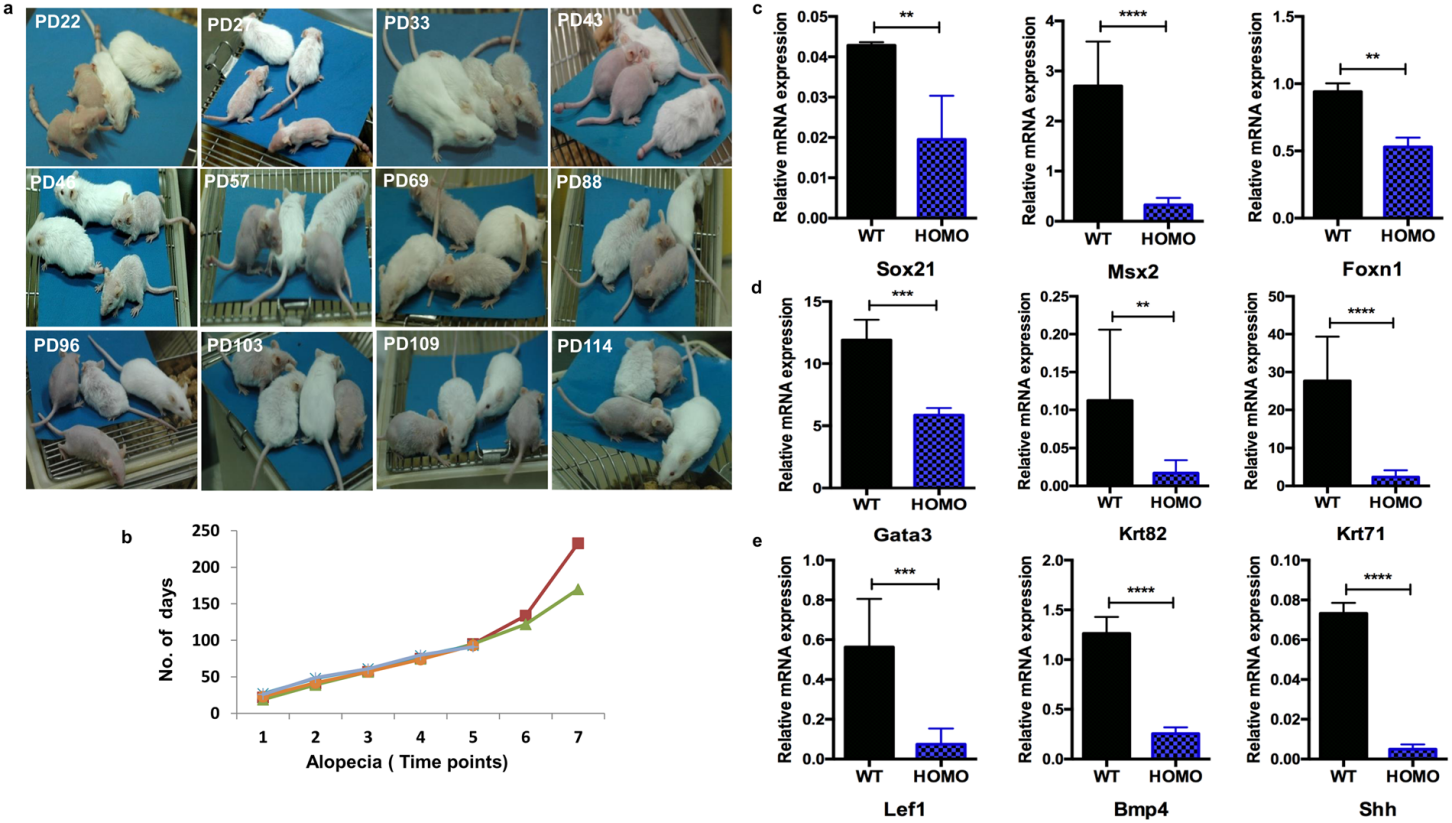
Cyclic alopecia in K14-sPLA_2_-IIA homozygous mice during various post-natal days. (**a**) Phenotypic appearance of WT and K14-sPLA_2_-IIA homozygous mice at different postnatal days with cyclic loss of hair and regain followed till six months. (**b**) Graphical representation of alopecia time points with respect to postnatal days of the K14-sPLA_2_-IIA homozygous mice. (**c**) Real time PCR gene expression analysis of Sox21, Msx2, Foxn1. (**d**) Gene expression analysis by Real time PCR of Gata3, Krt82 and Krt71. (**e**) Gene expression profiling of Lef1, BMP4, Shh. The relative quantification is with respect to expression level of β-actin. (WT-Wild type Homo- K14-sPLA_2_-IIA homozygous mice, n = 3 mice/genotype, PD-Postnatal days, Data are presented as mean ± SD. **P < 0.005, ***P < 0.001, ****P < 0.0001).

The new-born K14-sPLA_2_-IIA homozygous pups could be easily distinguished at PD3 as compared to wild type control littermate by their short and wavy whiskers. We followed the K14-sPLA_2_-IIA homozygous mice from birth (PD1) to up to six months (PD180) and recorded (photographed) the hair growth patterns over time at every alternate day. The hair loss began from PD18 and complete hair loss was observed at PD22 (Fig. 4a). Further, the hair regrowth was started at PD27 that partially covered the body by PD33 (Fig. 4a). However, the hair of K14-sPLA_2_-IIA homozygous mice was short compared to smooth and shiny hair of wild type control littermate. This successive cycle of hair growth and loss was occurring repetitively after 18–22 days up to 6–8 months (Fig. 4b). This cyclic alopecia was observed in both male and female mice. In addition, we observed permanent alopecia in K14-sPLA_2_-IIA homozygous mice starting at six months age till their survival (One year, Data not shown).

### Aberrant hair shaft differentiation mediated through deregulated expression of signalling modulators

We further investigated whether this cyclic phenomenon relies on the deregulation of known molecules or is there an unexplored novel mechanism. The expression levels of known genes such as Sox21, Msx2, Zdhcc13 and Foxn1 were analysed. Real time PCR data showed significant downregulation of Sox21, Msx2 and Foxn1 mRNA expression as compared to wild type control littermate (Fig. 4c). However, we did not observe any significant difference in expression level of Zdhcc13 and Gsdma3 mRNA (Supplementary Fig. S3). Importantly, Sox21 acts as a regulator of keratins expression, that is necessary for the formation of IRS (Inner root sheath) and anchoring of hair shaft. We examined the status of Gata3, Krt82 and Krt71 by real time PCR analysis. We found significant down-regulation of Gata3, Krt82 and Krt71 expression that suggest an aberrant differentiation of IRS and hair shaft precursor cells in K14-sPLA_2_-IIA homozygous mice (Fig. 4d). To understand the molecular mechanisms underlying impaired differentiation of the matrix cells, we checked the level of BMP4, Shh and Lef1, which are known to regulate the matrix cells proliferation and generation of precursors for IRS and hair shaft formation. We observed significant downregulation of BMP4, Lef1 and Shh in K14-sPLA_2_-IIA homozygous mice skin (Fig. 4e). These data suggested that deregulated signalling in matrix cells may lead to defects in differentiation of IRS and formation of hair shaft.

### Delayed wound healing response in K14-sPLA_2_-IIA homozygous mice

**Figure 5:**
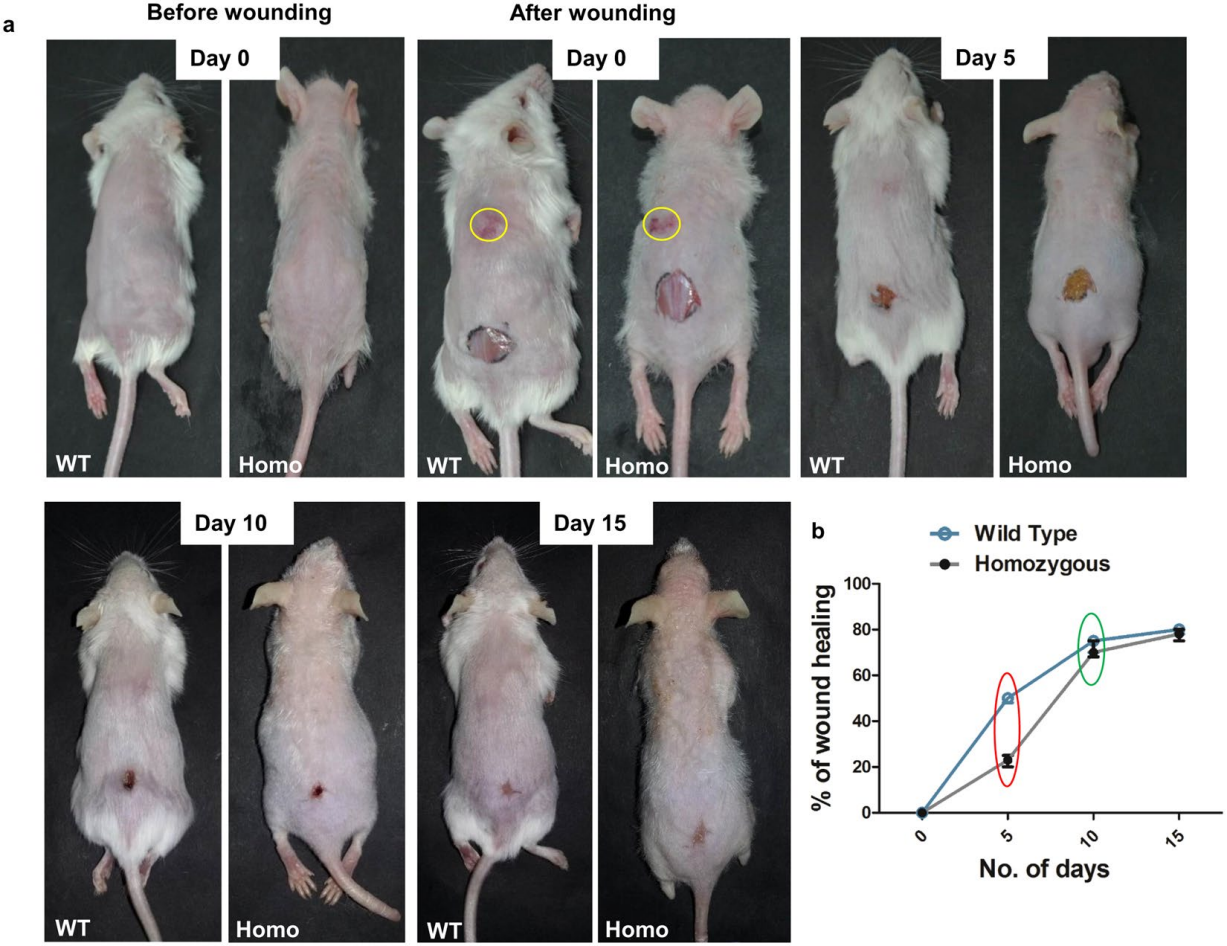
Impaired initial response to wounding in K14-sPLA_2_-IIA homozygous mice skin. (**a**) Photographs of WT and K14-sPLA_2_-IIA homozygous mice at Day 0 (before wounding) and at Day 0 (after wounding). The healing process was recorded by photographs at various days and representative images are mentioned as Day 5, Day 10 and Day 15. (**b**) Graphical representation of comparative wound recovery during healing process. Red circle indicates defects in initial healing response in K14-sPLA_2_-IIA homozygous mice. Green circle indicates no significant difference in time periods for complete wound recovery during later stage. (WT-Wild type Homo- K14-sPLA_2_-IIA homozygous mice, n = 3 mice/genotype, PD-Postnatal days).

Hair follicle stem cells contribute during epidermal regeneration after wounding. However, we observed drastic depletion of hair follicle stem cells in K14-sPLA_2_-IIA homozygous mice, which led us to evaluate wound healing response in K14-sPLA_2_-IIA homozygous mice. Scratch wounds were made on upper region at midline (yellow circle) (Fig. 5a) whereas, full thickness wounds (8 mm) were made on lower region of dorsal skin at PD49, that were monitored to assess the macroscopic healing defects (Fig. 5a). Our data showed impaired initial healing response in K14-sPLA_2_-IIA homozygous mice at day 5 (red circle) (Fig. 5b). However, the pace of wound recovery accelerated after 5 to 6 days and fully recovered at the same time of wild type control littermate (green circle) (Fig. 5b). To further investigate, whether the impaired wound healing response is due to the poor nutrition, we have checked the levels of serum albumin, Vitamin C, Vitamin D and Zinc from the serum. Our data showed that there is no significant difference in the levels of serum albumin, Vitamin C, Vitamin D and Zinc in the serum of the K14-sPLA_2_-IIA homozygous mice as compared to wild type control littermate (Supplementary Fig. S4). These results confirmed that the initial defects in the wound healing response may not be due to the poor nutrition. Thus, it demonstrates that overexpression of sPLA_2_-IIA delays initial response to wounding; however, the complete wound was filled at the same time point as compared to wild type control littermate.

## Discussion

The regenerative capability of adult tissue relies on activity of long-lived tissue specific stem cells. The cyclic activation of quiescent hair follicle stem cells depends on signalling cues present in the surrounding microenvironment. Involvement of different growth factors including EGF, TGFs and FGFs is well established in regulation of epidermal stem cells proliferation and fate determination^11^. As previously shown, deregulated release of Hb-EGF in mutant mice expressing soluble proHb-EGF induced epidermal hyperproliferation and displayed hyperplasia^31^. Overexpression of TGFα in transgenic mice epidermis demonstrates aberrant keratins expression, hyperproliferation and development of papillomas^32^. Further, expression of a dominant-negative FGF receptor mutant in transgenic mice under the control of Keratin 10 (K10) promoter induced epidermal hyper-thickening and altered keratinocytes differentiation^33^. Similarly, K14-sPLA_2_-IIA homozygous mice showed alterations in the hair follicle cycling, that are stuck in the anagen like phase of hair follicle and enhanced proliferation in various compartments of epidermis leading to epidermal hyperplasia. The sPLA_2_-IIA is known to enhance the EGF signalling in astrocytoma cells^34^ and continuous expression of epidermal growth factor prevents entry of hair follicle into the regression phase (Catagen) of hair cycle^15^. In agreement, our data of increased proliferation and hair follicle stuck at anagen like phase may be due to sPLA_2_-IIA mediated enhanced EGF signalling. Precisely, as EGF receptors are known to be expressed in the cells of outer root sheath of the hair follicle, and the proliferative response generated by upregulated mitogens (Hb-EGF and EPGN) directly suggest the involvement of catalytic independent activity of sPLA_2_-IIA in modulating EGFR signalling, which is not the case for the other group of secretory phospholipases. An aberrant signalling mediated hyperproliferation of the HFSCs resulted in loss of quiescence and exhaustion of stem cell pool. Smad4 deletion in mouse epidermis inhibits programmed regression of the hair follicles and exhibits progressive alopecia with depletion of hair follicle stem cells^26, 35^. Also, activation of mTOR by Wnt overexpression resulted in depletion of the hair follicle stem cells and accelerates hair loss and aging^36^. In agreement, our results showed hyperproliferation in various compartments of the hair follicle, that may lead to loss of quiescence and exhaustion of the hair follicle stem cells and development of alopecia.

Further, the contribution of the hair follicle stem cells in wound re-epithelialisation following injury is well established. Initially, the bulge-derived transient amplifying cells rapidly respond to the injury that contribute in wound repair and subsequently replaced by IFE derived cells over several weeks^37^. In contrast, partial ablation of the hair follicle stem cells by diphtheria toxin does not delay healing process following wounding thereby, suggesting that the bulge cells are dispensable for wound re-epithelialisation^38^. Epidermal hyperproliferation induced by deficiency of Jun-B resulted in delayed wound healing response^39^. Further, Runx1 deletion in mice showed delay in the activation of the HFSCs, which are further activated by skin injury^40^. Importantly, it has been shown that the bulge, hair follicle stem cells contribute to the process of wound healing and start migrating towards the wounded area within 24 hours, which suggests the involvement of the hair follicle stem cells in the initial process of wound healing^6, 37, 41, 42^. Our data of K14-sPLA_2_-IIA homozygous mice suggested that delay in initial response to wounding is likely due to the absence of immediate progenitor of the hair follicle stem cells, that sense the injury and effectively contributes towards initial healing response. Further, in agreement with the previous reports, IFE derived cells may take over the function and efficiently heal the wounded area at later time points.

Moreover, various studies have shown the phenomenon of permanent or cyclic alopecia in different genetic backgrounds of mice. The mice expressing dominant negative mutant of EGFR display severe alopecia^24^. Importantly, mice overexpressing human group II PLA_2_ displayed epidermal hyperplasia and complete alopecia^43^. Similarly overexpression of Pla2g2f in mice epidermis showed psoriasis like epidermal hyperplasia and alopecia^44^. Genetic disruption of the Sox21 and Msx2 resulted in defects in anchoring of hair shaft^25^ and aberrant hair shaft differentiation^28^ respectively. However, the level of Sox21 remained unaltered in Msx2 null background suggesting independent mode of action of both the molecules in the development of cyclic alopecia. We observed the downregulation of Sox21 and Msx2 in the K14-sPLA_2_-IIA homozygous mice suggesting that sPLA_2_-IIA may function upstream of both the factors and altering their expression. Further, mutation mediated downregulation of Zdhhc13 in mice exhibits epidermal hyperproliferation, abnormal hair follicle growth and cyclic alopecia at early telogen^45, 46^. However, we did not observe any alteration in Zdhhc13 level suggesting that the development of cyclic alopecia in K14-sPLA_2_-IIA homozygous mice is independent of cornifelin deficiency mediated cyclic alopecia as shown in Zdhhc13 mutant mice.

**Figure 6:**
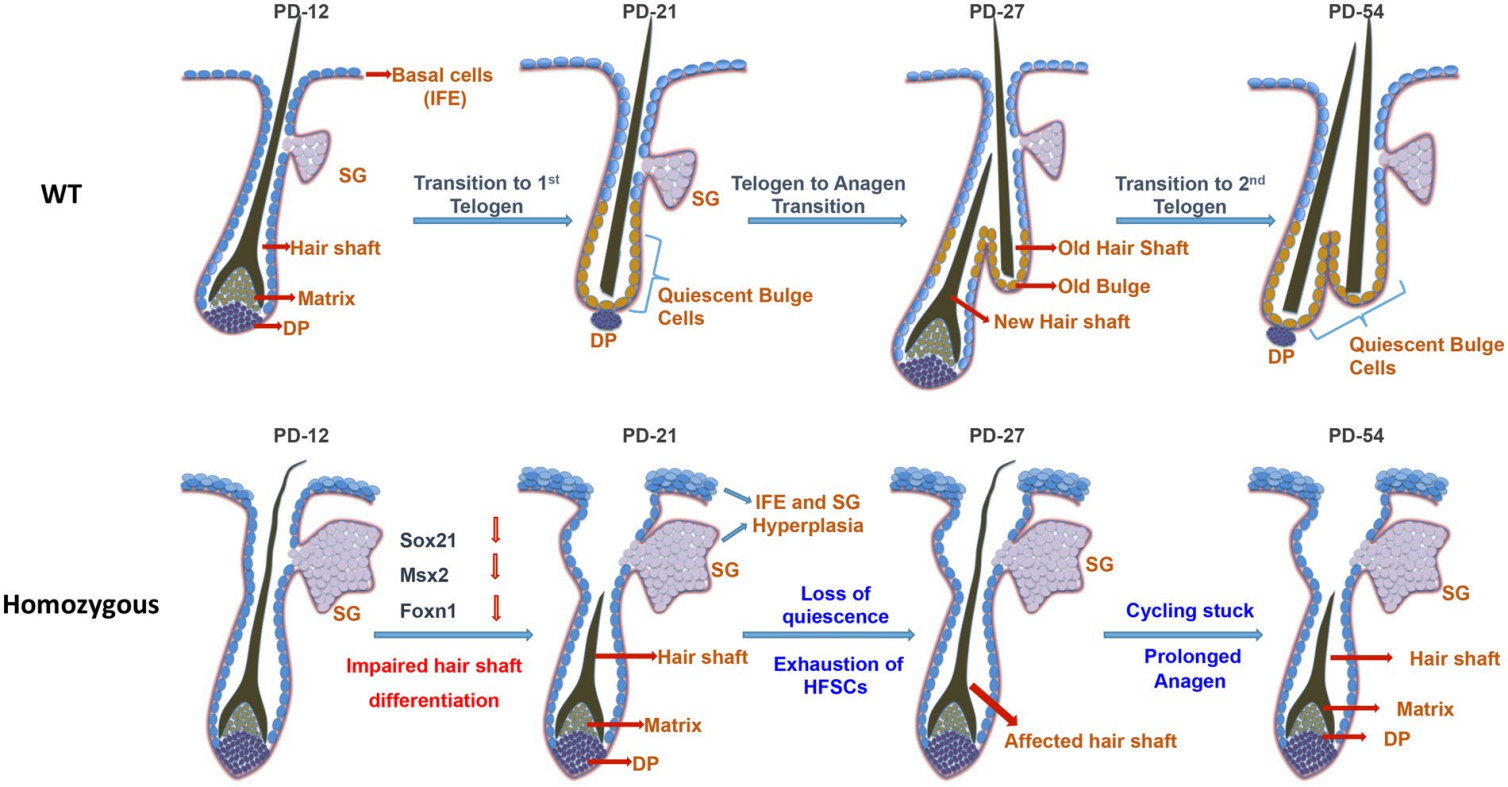
Proposed model of impaired hair follicle cycling in K14-sPLA_2_-IIA homozygous mice. **WT-** Graphical representation of hair follicle cycling in wild type mice from hair follicle morphogenesis to second telogen at PD49, which represented by presence of club hair and subsequent progression to the next hair cycle. **Homozygous-** Graphical representation of hair follicle cycling in K14-sPLA_2_-IIA homozygous mice represented by abnormal morphology of hair follicle with presence of affected hair shaft. (WT-Wild type Homo- K14-sPLA_2_-IIA homozygous IFE: inter follicular epidermis, SG: sebaceous gland, DP: dermal papilla, PD-Postnatal days).

Importantly, the proliferation and fate determination of matrix cells to differentiate in the IRS and hair shaft is governed by various signalling modulators such as BMP4, Lef1 and Shh. Specifically, BMP4 is expressed in the hair matrix cells and dermal papillae and is known to be involved in differentiation of the hair matrix cells to precursors cells required for hair shaft formation. Our results of gene expression study are in agreement with the previous study of sPLA_2_-X overexpressing transgenic mice, that showed down regulation of BMP4, Shh, Lef1, Foxn1 and Gata3 during development of cyclic alopecia^47^. Together, we found that overexpression sPLA_2_-IIA enhances the terminal epidermal differentiation however, the differentiation of matrix cells to produce precursors of the IRS and hair shaft is severely affected. This is likely due to the deregulated expression of keratin gene regulators such as Sox21 and matrix cells differentiation to produce hair shaft. Overall, these results suggest that sPLA_2_-IIA may affect differentiation of matrix cells. With regards to our finding, we have proposed the model for comparative hair follicle cycling (Fig. 6). Altered hair follicle cycling in K14-sPLA_2_-IIA homozygous mice represented by abnormal morphology of hair follicle with the presence of affected hair shaft. The hair follicle cycling does not progress through the first hair cycle, as the hair follicles are being stuck in anagen like stage during the initiation of first hair follicle cyling process. Further at PD28, hair follicle is represented by presence of affected hair shaft due to downregulated expression of Sox21, Msx2 and Foxn1, which are known to be involved in the IRS differentiation. Moroever, downregulation of signalling modulators such as Bmp4, Shh and Lef1 is observed in K14-sPLA_2_-IIA homozygous mice. Together, downregulated expression of hair shaft differentiation regulators may lead to the development of alopecia in K14-sPLA_2_-IIA homozygous mice.

In conclusion, we showed that sPLA_2_-IIA mediated increased proliferation results in the complete exhaustion of the HFSCs and development of alopecia due to aberrant expression of hair shaft differentiation regulators. The cyclic growth of hair and complete re-epithelialization of epidermis after wounding in absence of the HFSCs explains the functional involvement of stem cells from other compartments of the epidermis. This further opens a new avenue to explore the role of stem cells of other compartments of the epidermis and their ability to differentiate in hair shaft producing cells. This study provides a conceptual evidence of functional redundancy within stem cells population that reside in various compartments of epidermis. Further, the non-catalytic activity of sPLA_2_-IIA may provide information to develop potential sPLA_2_-IIA inhibitors for effective inhibition of sPLA_2_-IIA activity during various patho-physiological conditions such as arthritis, inflammation and cancer.

## Materials and Methods

### Transgenic mice and genotyping

K14-sPLA_2_-IIA mice were a gift from Dr. Rita Mulherkar^22^. The hemizygous K14-sPLA_2_-IIA was crossed to hemizygous K14-sPLA_2_-IIA mice to obtain the homozygous mice for the experiments. The K14-sPLA_2_-IIA hemizygous and homozygous transgenic mice were obtained based on phenotype and PCR genotyping as described previously^22^. Mice were sacrificed at various postnatal ages. Animal experimental study was approved by ACTREC’s Institutional Animal Ethics Committee (IAEC), and all the experiments were performed in accordance with the approved guidelines and regulations.

### Histology, Immunostaining and tail whole mount assay

Mice skin tissue (dorsal and tail skin) at various postnatal day ages were collected and fixed by neutral buffered formalin (NBF) or directly embedded in OCT compound (Tissue-Tek) and frozen. Immunofluorescence assays (IF) and Immunohistochemical analysis (IHC-P) was performed as previously described^7^. Paraffin embedded tissue blocks were sectioned and Haematoxylin and Eosin staining (H&E) was performed to analyze the phase of the hair follicle cycle. Immunofluorescence assays (IF) and Immunohistochemical analysis (IHC-P) was performed on OCT frozen tissue and Paraffin embedded block respectively^23^. Tail whole mount was performed as described previously^48^. Briefly, tail skin was incubated in 5 mM EDTA followed by separation of epidermal sheet from dermis followed by fixing with 2% formaldehyde for 10 minutes. Nile red was used to stain the sebocytes of sebaceous gland and confocal microscopy was used for image acquisition. Primary antibodies used such as: CD34 (1:100, BD Pharmingen); α-6 integrin (1:100, BD Pharmingen); BrdU (1:250, Abcam); Ki67 (1:100; Novocastra); Filaggrin (1:1000, Abcam); Loricrin (1:1000, Abcam); K10 (1:1000, Abcam); Lrig1 (1:500, R&D systems) and Sox9 (1:500, Merck Millipore).

### Fluorescence activated cell sorting analysis

Dorsal skin of wild type control littermate and K14-sPLA_2_-IIA homozygous mice at PD21 and PD49 was harvested and scrapped for fat removal, followed by overnight incubation in 0.25% trypsin at 4 °C. FACS experiments were performed as described previously^7^. Single cell suspension was obtained by first passing through 70 μm and then 40 μm cell strainers (BD Biosciences). Cells were stained with trypan blue and the hematocytometer chamber was used to count the cells. Further cells were stained by using the hair follicle stem cells markers: CD34-Biotin (eBiosciences), Streptavidin-APC (BD Pharmingen), and anti–α6-integrin-PE (BD Pharmingen). After washing cells were subjected to FACS acquisition using a FACS Aria and data was analyzed by using FACS DiVa software (BD Biosciences).

### Real-time quantitative PCR

Total RNA was extracted from mouse epidermis by using the Absolutely RNA Miniprep Kit (Agilent Technologies). 2 μg of total RNA was reverse transcribed using cDNA synthesis kit (Invitrogen, Carlsbad, CA) as per the manufacturer instructions. Quantitative PCR (q-PCR) was performed by using the SYBR Green (Invitrogen) as per the manufacturer’s instructions. Gene expression was normalized to β-actin. The relative expression levels of mRNAs were calculated by relative quantification method with respect to β-actin. For primers sequences please refer supplementary information.

### Wound healing assay

K14-sPLA_2_-IIA homozygous mice and control mice of 49 days were anaesthetized with isofluorane by inhalation for 30–60 seconds. After hair removal from the dorsal surface, single 8 mm full thickness excision skin wound on the midline was created. During the healing periods, recovery of the wounds was photographed. The wounded tissue was collected, OCT blocks and Paraffin blocks were prepared. Histological sections were stained with hematoxylin and eosin on paraffin blocks.

### Quantification of nutritional parameters from serum

Wild type and K14-sPLA_2_-IIA homozygous mice were starved for eight hours before collecting the blood. Serum was separated by centrifugation at 6000 rpm for 10 mins. Further, ascorbic acid was quantified by using ascorbic acid assay kit (ab65656) and zinc was quantified by using zinc quantification kit (ab102507) as per the manufacturer’s instructions. The nutritional parameters such as serum albumin, serum glucose, triglyceride, Sodium, Phosphorus, Chloride, total protein, Calcium and potassium was quantified by using Siemens’s Dimension EXL with LM, automated biochem analyzer and Vitamin B12 and Vitamin D 25-OH was quantified by using Abbott’s architect plus i1000SR as per the manufacturer’s instructions.

### Statistical analysis

Statistical significance was calculated to make the comparison between two groups by using the unpaired two-tailed student’s t-test with GraphPad Prism 5 for the data obtained from the measurement of IFE thickness, flow cytometry, Real time PCR, counting of hair follicle stem cells in bulge and nutritional parameters. Data represented with error bar indicating the mean ± SD of the mean: *P < 0.05, **P < 0.005, ***P < 0.0001.

## Electronic supplementary material

Supplementary Information
