# Publisher Correction: Secretory phospholipase A_2_-IIA overexpressing mice exhibit cyclic alopecia mediated through aberrant hair shaft differentiation and impaired wound healing response

**DOI:** 10.1038/s41598-020-62012-z

**Published:** 2020-03-13

**Authors:** Gopal L. Chovatiya, Rahul M. Sarate, Raghava R. Sunkara, Nilesh P. Gawas, Vineet Kala, Sanjeev K. Waghmare

**Affiliations:** 1 Stem Cell Biology Group, Waghmare Lab, Cancer Research Institute, Advanced Centre for Treatment Research and Education in Cancer (ACTREC), Tata Memorial Centre, Kharghar, Navi Mumbai, 410210 MH India; 2 Homi Bhabha National Institute, Training School Complex, Anushakti Nagar, Mumbai, 400085 India

Correction to: *Scientific Reports* 10.1038/s41598-017-11830-9, published online 14 September 2017

The original version of this Article contained an error in the Abstract.

“In addition, sPLA_2_-IIA induced hyperproliferation leads to compl pathological conditions including various cancers. Here ete exhaustion of hair follicle stem cell pool at PD28 (Postnatal day).”

now reads:

“In addition, sPLA_2_-IIA induced hyperproliferation leads to complete exhaustion of hair follicle stem cell pool at PD28 (Postnatal day).”

This error has now been corrected in the HTML and PDF versions of this Article.

