# Supplementary material for: Secretory phospholipase A2-IIA overexpressing mice exhibit cyclic alopecia mediated through aberrant hair shaft differentiation and impaired wound healing response
Source: Sci Rep. 2017 Sep 14;7:11619. doi: 10.1038/s41598-017-11830-9 (PMC5599634; doi:10.1038/s41598-017-11830-9)
Supplement: Supplementary file 1 — Supplementary Information [file 41598_2017_11830_MOESM1_ESM.pdf]

Supplementary Information:

Secretory phospholipase A_2-IIA overexpressing mice exhibit cyclic alopecia mediated through aberrant hair shaft differentiation and impaired wound healing response

Gopal L Chovatiya[1,2#], Rahul M Sarate[1,2#], Raghava R Sunkara[1,2], Nilesh P Gawas[1], Vineet Kala[1] and Sanjeev K Waghmare[1,2*]

[1] Stem Cell Biology Group, Waghmare Lab, Cancer Research Institute, Advanced Centre for Treatment Research and Education in Cancer (ACTREC), Tata Memorial Centre, Kharghar, Navi Mumbai 410210, MH, India.

[2] Homi Bhabha National Institute, Training School Complex, Anushakti Nagar, Mumbai 400085, India

# Authors contributed equally

***Corresponding Author:** Sanjeev K Waghmare, Stem Cell Biology Group, Waghmare Lab, Cancer Research Institute, Advanced Centre for Treatment Research and Education in Cancer (ACTREC) Tata Memorial Centre, Kharghar, Navi Mumbai 410210, MH, India, E-mail: swaghmare@actrec.gov.in

Supplementary Figure S1

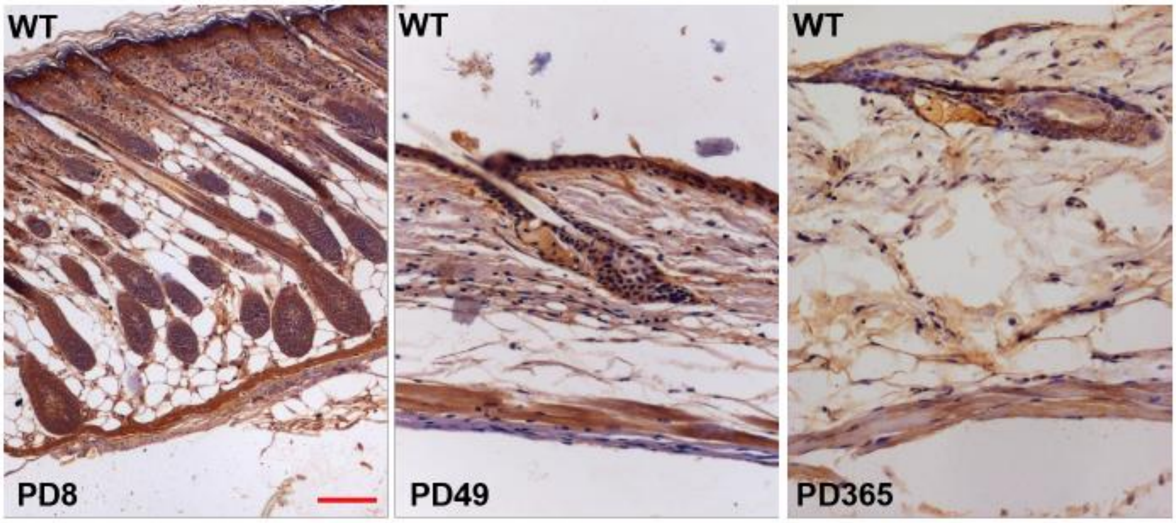

Supplementary Fig S1: Immunohistochemical staining of $sPLA_2$-IIA on dorsal skin sections of wild type mice at PD8, PD49 and PD365. (WT- Wild type n=4 mice/genotype. Scale bar: 100 μm)

Supplementary Figure S2

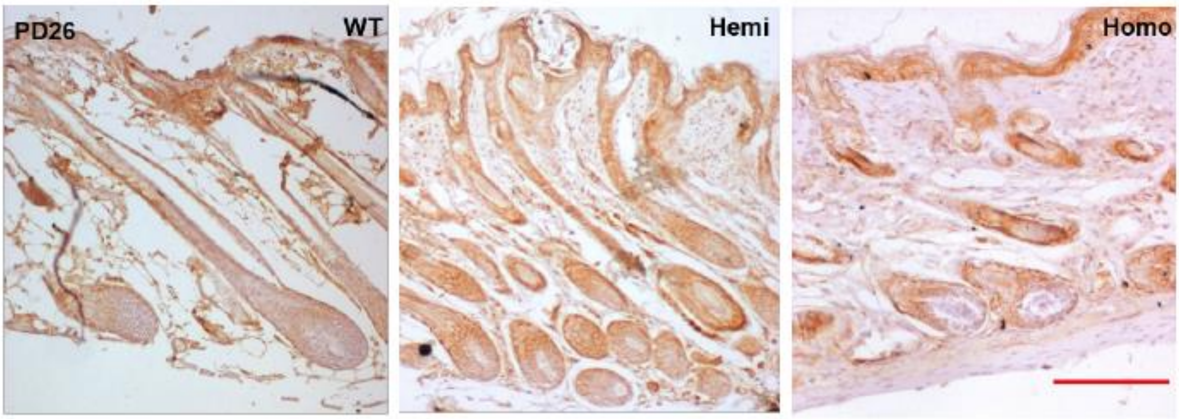

Supplementary Fig S2: Immunohistochemical staining of active β-catenin at PD 49 in dorsal skin sections of WT and K14-$sPLA_2$-IIA hemizygous and homozygous mice. (WT- Wild type, Hemi- hemizygous mice and Homo- K14-$sPLA_2$-IIA homozygous mice. n=4 mice/genotype. Scale bar: 100 μm)

Supplementary Figure S3

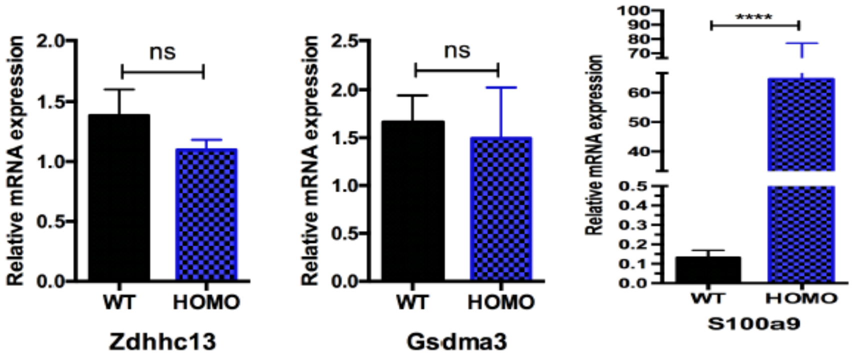

Supplementary Fig S3: Real time quantitative PCR analysis of Zdhhc13, Gsdma3 and S100a9 mRNA from epidermal cells of WT and K14-sPLA$_2$-IIA homozygous mice. (WT- Wild type and Homo- K14-sPLA$_2$-IIA homozygous mice. n=4 mice/genotype. Data are presented as mean ± SD. **P<0.005, ***P<0.0001)

Supplementary Figure S4

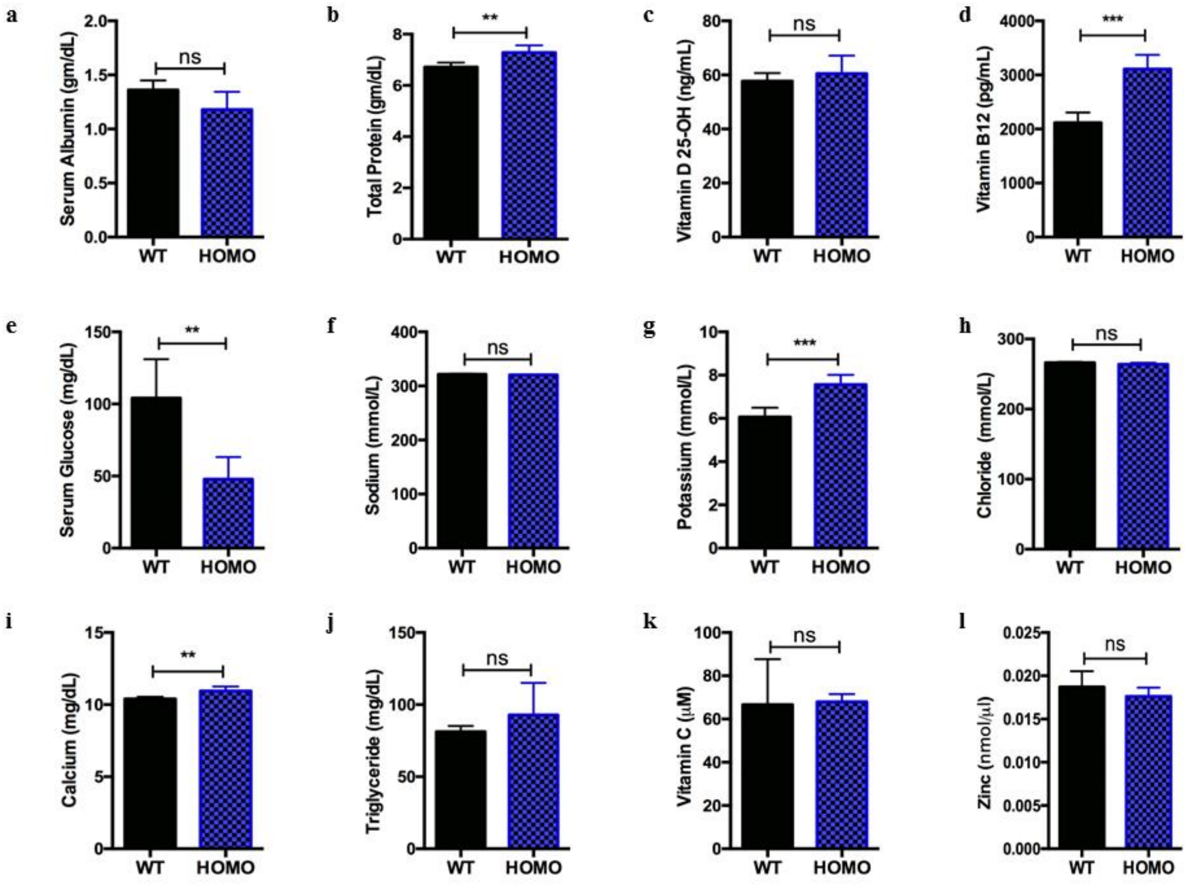

Supplementary Fig S4: Quantification of nutritional parameters such as serum albumin, total protein, Vitamin D 25-OH, Vitamin B12, serum glucose, Sodium, Potassium, Chloride, Calcium, Triglyceride, Vitamin C and Zinc. (WT- Wild type and Homo- K14-sPLA$_2$-IIA homozygous mice. n=4 mice/genotype. Data are presented as mean ± SD. **P<0.005, ***P<0.0001)

Supplementary Figure S5

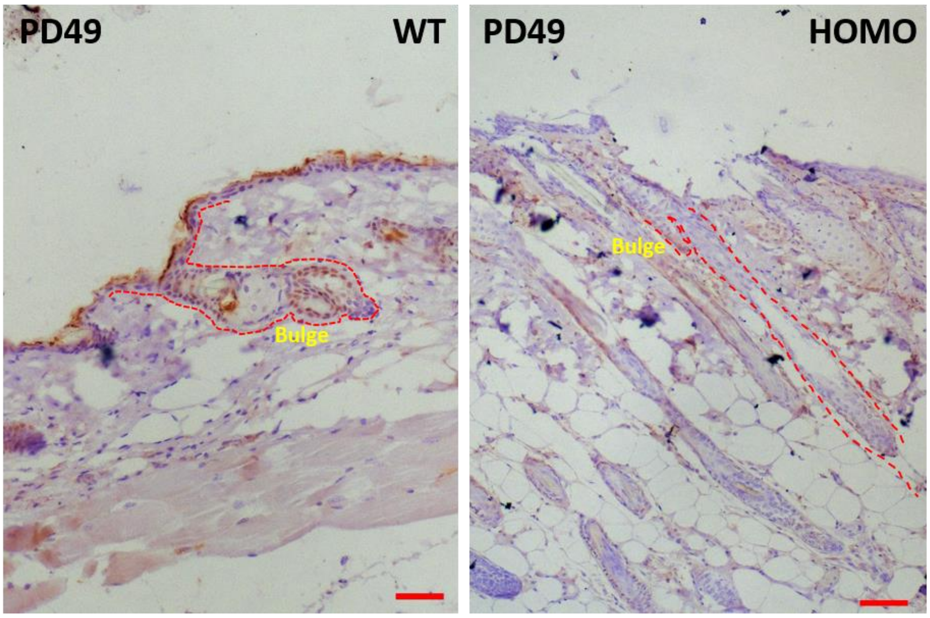

Supplementary Fig S5: Immunohistochemical staining of Sox9 at PD 49 in dorsal skin sections of WT and homozygous mice. (WT- Wild type and Homo-K14-sPLA$_2$-IIA homozygous mice. n=3 mice/genotype. PD- postnatal day, Scale bar: 100 μm)

Primers sequences used for Real time PCR

Gene	Forward Primer 5’--->3’	Reverse Primer 5’--->3’
Sox21	CCTGGGCAGCGTGGCGGA	CAGACTGCGGGAAGAAGACG
Msx2	AACACAAGACCAACCGGAAG	CGCTCTGCTATGGACAGGTA
Krt82	TCTATGGGGCTGAAGACCAG	GGTGGCTTTGAAGAAATGA
Krt71	TCAGATCCAGTCCCACATCA	GTACAGGGCCTCAGCTTCAG
Zdhhc13	GACTGGACGCTGCATAGGTT	TGGCACAATGATTTGACCAG
Gata3	TTATCAAGCCCAAGCGAAG	TGGTGGTGGTCTGACAGTTC
Shh	ACCCCGACATCATATTTAAGGA	TTAACTTGTCTTTGCACCTCTGA
Foxn1	TGACGGAGCACTTCCCTTAC	GACAGGTTATGGCGAACAGAA
S100a9	CACCCTGAGCAAGAAGGAAT	TGTCATTTATGAGGGCTTCATTT
Lef1	GCCACCGATGAGATGATCCC	TTGATGTCGGCTAAGTCGCC
BMP4	GCCCTGCAGTCCTTCGCTGG	CTGACGTGCTGGCCCTGGTG
